# Heteroatom-doped MXene quantum dots for selective transition metal ion sensing: from atomic-level design to intelligent and deployable platforms

**DOI:** 10.1039/d6ra02353c

**Published:** 2026-06-04

**Authors:** Enas Daoud, Bakr Imad Najm, Omayma Salim Waleed, Maharshikumar B. Shukla, Rekha M. M., Y. Sasikumar, Vipasha Sharma, Ahmed Aldulaimi, Sharmin Smaeilpour

**Affiliations:** a Faculty of Allied Medical Sciences, Hourani Center for Applied Scientific Research, Al-Ahliyya Amman University Amman Jordan; b College of Pharmacy, Department of Pharmaceutical Sciences, AL-Turath University Baghdad Iraq; c Department of Anesthesia Techniques, Health and Medical Techniques College, Alnoor University Mosul Iraq; d Department of Chemistry, Faculty of Science, Gokul Global University Sidhpur Gujarat India; e Department of Chemistry and Biochemistry, School of Sciences, JAIN (Deemed-to-be University) Bangalore Karnataka India; f Department of Chemistry, Sathyabama Institute of Science and Technology Chennai Tamil Nadu India; g Department of Biotechnology, University Institute of Biotechnology, Chandigarh University Mohali Punjab India; h Faculty of Pharmacy, Al-Zahrawi University Karbala Iraq; i Young Researchers and Elite Club, Islamic Azad University Tehran Branch Tehran Iran sharminsmaeilpour@gmail.com

## Abstract

Heteroatom-doped MXene quantum dots (MQDs) have emerged as promising fluorescent nanoplatforms for the selective detection of transition metal ions such as Fe^3+^, Cu^2+^, Zn^2+^, and Mn^2+^. Their tunable electronic structure, high quantum yield, and versatile surface chemistry enable precise modulation of optical properties and binding interactions with metal ions. This review provides a comprehensive overview of recent advances in the design and application of heteroatom-doped MQDs for transition metal ion sensing. Particular emphasis is placed on atomic-level engineering strategies, including dopant–host electronic coupling, defect–dopant synergy, single-atom doping, selective functionalization at edge *versus* basal-plane sites, and multi-element doping (*e.g.*, S, P, B, and halogens). These structural modifications enable tailored control over charge distribution, redox activity, and coordination environments, thereby improving sensitivity and ion selectivity. Beyond conventional fluorescence quenching mechanisms, emerging sensing strategies are also discussed, including ratiometric detection, stimuli-responsive probes, multimodal sensing systems integrating optical, electrochemical, and visual signals, and logic-gated or data-assisted sensing approaches designed to improve analytical reliability in complex matrices. Representative sensing behaviors are highlighted, such as redox-mediated quenching for Fe^3+^ and Cu^2+^, fluorescence enhancement for Zn^2+^, and dual-emission ratiometric recognition for Mn^2+^. Finally, current challenges—including synthesis scalability, selectivity in competitive environments, matrix interference, and translation toward deployable sensing devices—are critically evaluated, and future directions for portable sensing platforms and intelligent analytical systems are discussed.

## Abstract 2

Heteroatom-doped MXene quantum dots offer atomically tunable electronic structures and surface sites for highly selective transition metal ion sensing.

## Graphical abstract

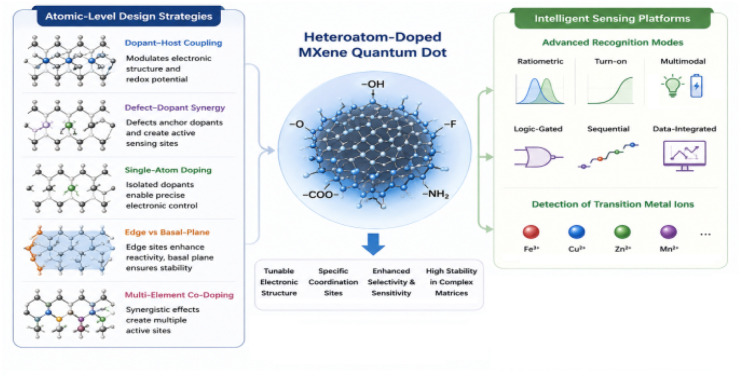

## 1. Introduction

Transition metal ions, including Fe^3+^, Cu^2+^, Zn^2+^, and Mn^2+^, play pivotal roles in environmental ecosystems, biological processes, and industrial applications.^1–3^ These ions are essential micronutrients for enzymatic functions, oxygen transport, and cellular signaling in living organisms, yet their dysregulation can lead to severe health issues such as neurodegenerative diseases, oxidative stress, and heavy metal poisoning.^4–7^ In environmental contexts, elevated concentrations from industrial effluents, mining activities, and agricultural runoff pose significant risks to water quality and biodiversity.^8,9^ Consequently, the selective and sensitive detection of these ions is imperative for environmental monitoring, food safety assessment, clinical diagnostics, and wastewater management. Traditional analytical techniques, such as atomic absorption spectroscopy (AAS), inductively coupled plasma mass spectrometry (ICP-MS), and electrochemical methods, offer high precision but are often hampered by high costs, complex instrumentation, and the need for sample pretreatment, limiting their applicability in real-time, on-site scenarios.^10–13^ Fluorescence-based sensors have emerged as promising alternatives due to their simplicity, rapid response, and compatibility with portable devices.^14,15^ However, conventional fluorescent probes, including organic dyes and semiconductor quantum dots (QDs), suffer from limitations such as photobleaching, toxicity, poor selectivity in complex matrices, and insufficient tunability for multi-ion discrimination.^16,17^

In this landscape, MXene quantum dots (MQDs)—ultrasmall, zero-dimensional derivatives of two-dimensional MXenes—have garnered substantial attention as next-generation fluorescent nanomaterials.^18,19^ MXenes, a family of transition metal carbides, nitrides, or carbonitrides with the general formula M_{*n*+1}_X_*n*_T_*x*_ (where M is a transition metal, X is carbon/nitrogen, and T_*x*_ denotes surface terminations), exhibit exceptional properties including high electrical conductivity, hydrophilic surfaces, and tunable bandgaps. When downsized to quantum dots *via* exfoliation or bottom-up synthesis, MQDs inherit these attributes while gaining quantum confinement effects, leading to bright photoluminescence (PL), high quantum yields (QYs), and excellent photostability. Unlike traditional QDs (*e.g.*, CdSe or InP), MQDs are composed of earth-abundant elements, offering biocompatibility and reduced toxicity, making them suitable for bioenvironmental applications.^20–22^ The rich surface chemistry of MQDs, characterized by abundant functional groups (–OH, –O, –F), enables facile modification for targeted sensing.

It is important to distinguish MQDs from their bulk or two-dimensional MXene counterparts. While bulk MXenes primarily exhibit metallic conductivity and layered structures with relatively uniform electronic behavior, MQDs possess ultrasmall dimensions typically below 10 nm. At this scale, strong quantum confinement and a high density of edge and defect sites significantly modify their electronic structure, surface chemistry, and photoluminescence properties. These size-dependent effects generate discrete energy states and enhanced surface reactivity, which are key factors enabling MQDs to function as highly sensitive platforms for fluorescence-based ion sensing.

Heteroatom doping represents a transformative strategy to enhance the sensing capabilities of MQDs. By incorporating non-metal elements such as nitrogen (N), sulfur (S), phosphorus (P), boron (B), or halogens into the MQD lattice, researchers can precisely modulate electronic structure, charge distribution, and surface reactivity.^23,24^ This atomic-level engineering shifts MQDs from passive emitters to active recognition platforms. For instance, N-doping introduces electron-donating states, enhancing PL efficiency and creating coordination sites for metal ions, while S-doping imparts redox-active properties for selective quenching. Co-doping (*e.g.*, N + S) synergistically amplifies these effects, enabling emergent behaviors like ratiometric responses.^23,25^ Such modifications address key challenges in transition metal ion sensing: achieving nanomolar sensitivities, discriminating between ions with similar redox potentials, and maintaining robustness in interferent-rich matrices.

The evolution of MQD-based sensors has progressed from basic intensity-based quenching to sophisticated paradigms. Early designs focused on static fluorescence modulation, where ion coordination disrupts radiative recombination, leading to “turn-off” signals. However, these are susceptible to environmental artifacts. Recent advancements emphasize ratiometric architectures, where dual-emission channels provide self-calibration, mitigating fluctuations in probe concentration or excitation intensity. Stimuli-responsive systems further introduce adaptability, allowing reversible “on–off” cycles in response to pH or competing analytes.^27,28^ Multimodal integration—combining optical, electrochemical, and visual outputs—enhances versatility, while logic-gated and sequential recognition encode chemical events as computational operations, suppressing false positives in multi-ion environments. Data-integrated platforms, leveraging machine learning for pattern analysis, foreshadow intelligent sensing ecosystems.^29,30^

Performance analyses underscore the efficacy of heteroatom-doped MQDs for specific ions. For redox-active Fe^3+^ and Cu^2+^, amino-functionalized Ti_3_C_2_ MQDs achieve quenching-based detection with limits of detection (LODs) in the low nanomolar range, suitable for environmental thresholds. Zn^2+^ sensing exploits “turn-on” enhancement *via* excited-state stabilization, offering selectivity over quenching-prone ions. Mn^2+^ detection benefits from dual-emission ratiometry, providing robust quantification in mineral waters. Emerging Nb_2_C-based MQDs expand material diversity, combining sensing with bioimaging.^31–33^ Selectivity engineering, *via* masking agents or differential signal patterns, addresses competitive binding, while portability trends—test strips and smartphone readers—facilitate field deployment.

Despite these strides, challenges persist. Synthesis scalability remains limited by batch variability and high-energy processes, hindering commercial viability. Fundamental selectivity boundaries arise from overlapping ion affinities, necessitating computational-guided design. Matrix robustness under variable conditions (*e.g.*, pH, salinity) demands further optimization. Future directions include IoT integration for continuous monitoring, biocompatible formulations for *in vivo* sensing, and multifunctional devices for simultaneous multi-ion analysis.^26–28^

This review examines heteroatom-doped MQDs as promising platforms for selective transition metal ion sensing. It integrates atomic-level design strategies—dopant–host coupling, defect synergy, single-atom precision, spatial doping control, and multi-element expansion—with advanced recognition paradigms, including ratiometric, stimuli-responsive, multimodal, logic-gated, sequential, and intelligent data-driven systems. Ion-specific performance for Fe^3+^, Cu^2+^, Zn^2+^, and Mn^2+^ is assessed, alongside selectivity enhancement in complex matrices and pathways to portable, deployable formats. Key challenges in scalability, matrix robustness, selectivity limits, and integration are evaluated, while future directions such as computational optimization, IoT networks, and bioanalytical multifunctionality are outlined. This work synthesizes recent high-impact progress to guide the evolution of MQD-based technologies in environmental monitoring, water quality, food safety, and biomedical applications. This review primarily focuses on advances in MXene quantum dots reported during the period 2021–2026, highlighting the most recent developments in heteroatom doping strategies and sensing applications, while selectively citing earlier studies to provide essential background for the field.

Several recent reviews and studies have examined MQDs from different perspectives. Previous works have mainly focused on multifunctional water remediation combining photocatalysis, electrocatalysis, and fluorescence sensing,^33^ as well as nitrogen-doped MQDs emphasizing mechanistic fluorescence, electrochemical, and electrochemiluminescence sensing platforms.^19,27^ Other studies have investigated engineered MQDs for energy-related applications such as micro-supercapacitors rather than selective sensing.^29^ In contrast, the present review provides a broader design-oriented framework that systematically correlates heteroatom doping beyond nitrogen, atomic-level structural engineering, and advanced recognition paradigms—including ratiometric, multimodal, and logic-gated sensing—specifically for selective transition metal ion detection. This integrated structure–mechanism–performance perspective distinguishes the scope of this review.

## 2. Atomic-level design strategies for heteroatom-doped MQDs: beyond conventional doping concepts

### 2.1. Dopant–host electronic coupling in MQDs: tailoring local electronic environments

The incorporation of heteroatoms into MQDs introduces profound modifications to their local electronic environments, extending far beyond simple bandgap tuning. At the atomic scale, dopant–host interactions alter charge density distributions, local work functions, and orbital hybridization patterns. Unlike bulk MXenes, MQDs exhibit pronounced quantum confinement and high edge-to-surface ratios, amplifying the electronic influence of individual dopant atoms. As a result, heteroatom doping in MQDs should be understood as a localized electronic coupling phenomenon rather than a homogeneous bulk modification.^34,35^

From a materials design perspective, the electronic role of dopants is governed by their electronegativity, valence configuration, and coordination preferences relative to the parent MXene lattice. Substitutional dopants can introduce localized donor or acceptor states, while interstitial or surface-anchored dopants may act as charge polarization centers. These effects lead to spatially heterogeneous electronic landscapes within a single MQD, creating regions with distinct redox potentials and electron affinity. Such heterogeneity is particularly relevant for transition-metal ion interactions, as it dictates preferential adsorption and coordination sites at the nanoscale.^36^

Importantly, dopant–host coupling in MQDs is strongly size-dependent. As MQD dimensions approach the exciton Bohr radius, even low dopant concentrations can dominate electronic behavior. This sensitivity necessitates precise control over dopant placement and concentration, shifting the design paradigm from average composition toward atomistic precision. Emerging synthetic approaches increasingly aim to regulate dopant-induced electronic anisotropy, enabling rational control over MQD reactivity without relying on post-synthetic functionalization.^37,38^ This atomic-level understanding forms the foundation for next-generation MQD design strategies.

**Fig. 1:**
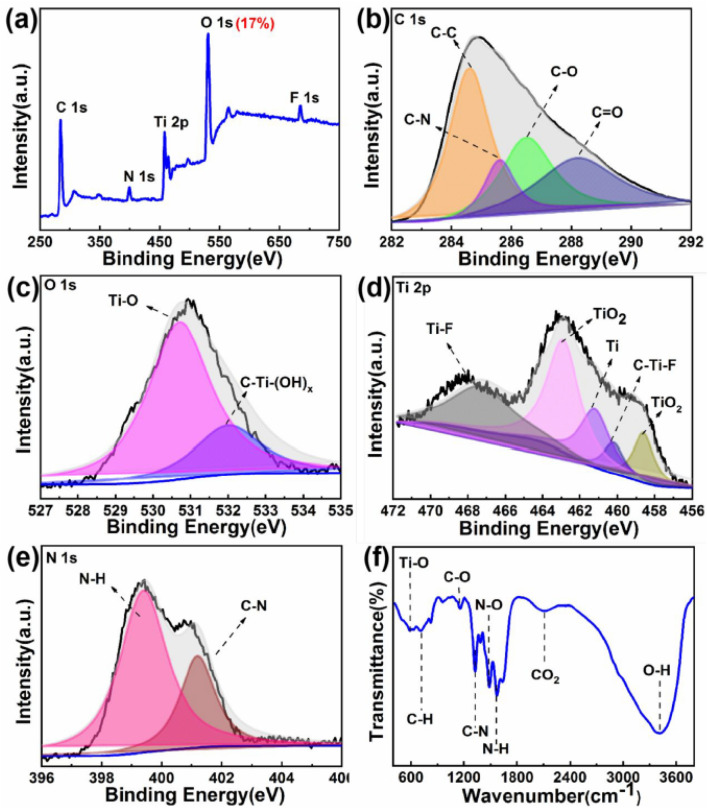
(a) XPS survey spectrum, (b) high-resolution C 1s, (c) O 1s, (d) Ti 2p, (e) N 1s, and (f) FT-IR spectra of N-Ti_2_C MQDs, revealing dopant–host electronic coupling and defect-stabilization effects through preservation of Ti–C bonding and nitrogen-induced localized electronic states. This figure has been reproduced from ref. 38 with permission from American Chemical Society, copyright 2021.

Panels (1a) and (1d) provide clear spectroscopic evidence that nitrogen incorporation modifies the electronic interaction between dopant atoms and the Ti_2_C host lattice. The XPS survey spectrum (Fig. 1a) shows a decrease in oxygen-related signals together with stronger Ti and C contributions, indicating that the Ti_2_C framework remains largely preserved during the doping process. This observation suggests that nitrogen incorporation occurs without extensive oxidation of the MXene structure. The Ti 2p spectrum (Fig. 1d) further supports this interpretation, where the dominant Ti–C peaks confirm the integrity of the Ti–C lattice, while only weak Ti–O features are observed. The preservation of Ti–C bonding together with the presence of nitrogen indicates that dopants interact electronically with the Ti_2_C lattice rather than forming separate oxide phases. Consequently, nitrogen atoms introduce new electronic states within the MQD structure, modifying the local charge distribution and strengthening dopant–host electronic coupling within the quantum-confined lattice.

### 2.2. Defect–dopant interactions: engineering active sites through coupled structural imperfections

In MQDs, intrinsic defects such as vacancies, edge terminations, and lattice distortions are not merely unavoidable imperfections but powerful design elements when coupled with heteroatom doping. Defect–dopant interactions can stabilize otherwise unfavorable dopant configurations and create synergistic active sites with enhanced chemical reactivity. The reduced dimensionality of MQDs magnifies these interactions, making defect engineering a central strategy rather than a secondary consideration.^39,40^

Atomic vacancies, particularly metal or carbon vacancies, can act as anchoring centers for heteroatoms, lowering the formation energy of doped structures. When a dopant occupies a vacancy-adjacent site, local coordination symmetry is disrupted, leading to asymmetric charge redistribution. This asymmetry can induce localized dipole moments and strain fields, both of which strongly influence surface chemistry.^41–43^ Such coupled defect–dopant motifs often exhibit electronic states within the bandgap, enabling fine control over charge transfer processes.

Edge defects play an even more prominent role in MQDs due to their high proportion relative to basal planes. Edge-selective doping strategies exploit the undercoordinated nature of edge atoms to introduce heteroatoms with minimal lattice disruption. The resulting edge-confined active sites combine structural flexibility with electronic tunability, offering a versatile platform for controlled interactions with external species.^40,44^ From a design standpoint, the deliberate coupling of defects and dopants represents a shift from defect minimization toward defect utilization, enabling the creation of function-specific MQDs with tailored atomic architectures.

Fig. 1b and c provide insight into how dopants interact with defect-rich regions formed during quantum dot generation. The reduced intensity of C–O components in the C 1s spectrum (Fig. 1b), together with the simplified O 1s profile dominated by Ti–O and C–Ti–(OH)_*x*_ bonds (Fig. 1c), indicates that defect-induced oxidation pathways are effectively suppressed. Given the high density of undercoordinated sites created as Ti_2_C MXenes are converted into MQDs, these observations suggest preferential dopant interaction with defect-adjacent sites, stabilizing them against further structural degradation.

The FT-IR spectrum in Fig. 1f, in conjunction with the N 1s spectrum (Fig. 1e), further supports the formation of coupled defect–dopant motifs. Vibrational signatures associated with C–N and N–H groups indicate that nitrogen-containing functionalities are selectively anchored at defect-prone surface regions. Rather than acting as passive surface terminations, these dopant-modified sites actively reshape the defect landscape, converting intrinsically reactive imperfections into electronically stabilized motifs. This behavior exemplifies defect utilization rather than defect elimination.

**Fig. 2:**
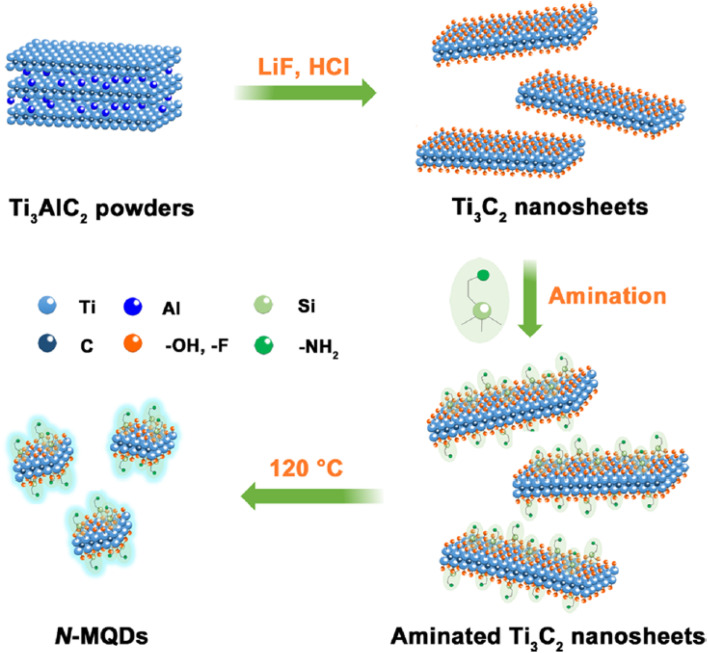
Schematic synthesis of N-doped MQDs from Ti_3_AlC_2_ highlighting defect formation and dopant incorporation during quantum cutting. This figure has been reproduced from ref. 44 with permission from Elsevier, copyright 2025.

Fig. 2 illustrates the synthetic pathway leading to the formation of N-doped MQDs from Ti_3_AlC_2_ MAX precursors and provides mechanistic insight into how defect–dopant coupling emerges during the process. The LiF/HCl etching step selectively removes Al layers, producing Ti_3_C_2_ MXene sheets rich in surface terminations and structural imperfections that act as chemically active sites. Subsequent ultrasonic exfoliation and chemical functionalization with APTES introduce nitrogen-containing groups that preferentially interact with undercoordinated atoms and vacancy-adjacent regions generated during MXene delamination. These defect-rich regions provide energetically favorable anchoring environments for dopant incorporation, enabling the stabilization of nitrogen functionalities within the evolving MQD structure. During the hydrothermal cutting process that converts aminated nanosheets into quantum dots, the high density of edges and lattice discontinuities further amplifies defect availability, promoting the formation of coupled defect–dopant motifs. As a result, nitrogen dopants become integrated within defect-proximal sites rather than randomly distributed on pristine lattice domains, illustrating how structural imperfections formed during MQD generation can be actively utilized to engineer chemically stable and electronically tunable active sites.

### 2.3. Single-atom and low-concentration doping regimes: precision engineering at the quantum limit

As MQDs approach ultrasmall dimensions, traditional concepts of bulk or even surface doping become inadequate. In this regime, single-atom or ultra-low-concentration doping emerges as a powerful strategy for precision engineering. A single heteroatom can dominate the electronic and chemical behavior of an entire MQD, effectively acting as an atomic-scale functional unit embedded within the host lattice. Single-atom doping introduces discrete electronic states that are highly localized yet strongly coupled to the quantum-confined host. These states can serve as well-defined charge trapping or transfer centers, offering unparalleled control over electronic dynamics.^45,46^

Unlike higher dopant concentrations, which may introduce disorder or nonradiative recombination pathways, single-atom dopants preserve structural integrity while enabling targeted functionality. This balance is particularly attractive for applications requiring high selectivity and reproducibility. From a synthetic standpoint, achieving single-atom doping in MQDs demands stringent control over precursor chemistry and reaction kinetics. Advances in bottom-up synthesis, precursor-limited growth, and *in situ* coordination control are making such precision increasingly feasible.^28,44^ The resulting MQDs challenge conventional compositional descriptors, as their properties cannot be averaged over dopant distributions. Instead, they necessitate atom-by-atom design logic, positioning single-atom-doped MQDs at the frontier of quantum materials engineering.

**Fig. 3:**
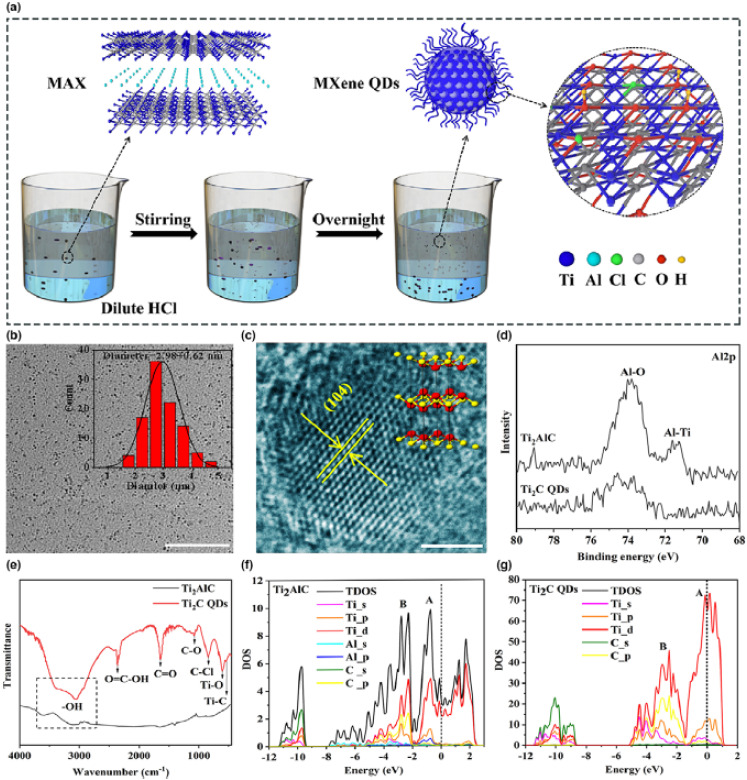
Structural and electronic characterization of Ti_2_CT_*x*_ MQDs. (a) Schematic of HCl-etching synthesis route. (b) TEM image and size distribution (∼3 nm). (c) HRTEM showing lattice spacing of MQDs. (d) XPS spectra confirming removal of Al and formation of Ti–C bonds. (e) FTIR revealing surface functional groups. (f and g) Density of states of Ti_2_AlC and Ti_2_C MQDs showing Ti-3d dominated states near the Fermi level. This figure has been reproduced from ref. 69 with permission from Wiley Online Library, copyright 2023.

Fig. 3 provides structural and electronic evidence illustrating how ultrasmall MQDs create a suitable platform for precision engineering in the single-atom or ultra-low-concentration doping regime. Panel (a) outlines the synthesis pathway, where selective etching of the Al layer from Ti_2_AlC generates Ti_2_CT_*x*_ MQDs decorated with surface functional groups. This process not only delaminates the layered precursor but also produces nanoscale domains enriched with chemically active surface sites. The TEM image in panel (b) shows highly dispersed MQDs with an average diameter of approximately 3 nm. At this dimension, the entire particle essentially operates within the quantum confinement regime, where the electronic structure becomes highly sensitive to atomic-scale perturbations. Consequently, the introduction of even a single heteroatom or a very small number of dopants can significantly influence the electronic behavior of the whole quantum dot, highlighting why such ultrasmall MQDs are promising hosts for atomically precise doping strategies.

Panels (c)–(g) further clarify the structural integrity and electronic characteristics that enable such precision engineering. The HRTEM image in panel (c) reveals well-defined lattice fringes corresponding to the (104) plane, indicating that the crystalline framework remains intact despite the extreme size reduction. Maintaining this structural order is essential because single-atom dopants interact strongly with the host lattice and their influence depends on well-defined local coordination environments. XPS spectra in panel (d) confirm the removal of Ti–Al bonds and the formation of Ti–C bonds, while FTIR analysis in panel (e) verifies the presence of surface functional groups such as –O and –OH. These groups can serve as coordination anchors that stabilize isolated heteroatoms or dilute dopant species. Finally, the density of states calculations in panels (f) and (g) show that Ti 3d orbitals dominate near the Fermi level, implying a high sensitivity of the electronic structure to local atomic modifications. Such sensitivity suggests that even a single dopant atom could introduce localized electronic states capable of modulating charge transfer dynamics. Nevertheless, it is important to note that while Fig. 1 establishes the structural and electronic framework conducive to single-atom doping, it does not directly visualize isolated dopant atoms; techniques such as HAADF-STEM or EXAFS would be required for definitive confirmation.

### 2.4. Edge *versus* basal-plane doping: spatial control of chemical reactivity

The spatial distribution of dopants within MQDs critically determines their chemical behavior. Edge and basal-plane doping represent two fundamentally distinct design approaches, each imparting unique structural and electronic characteristics. Given the dominance of edge sites in MQDs, selective edge doping offers a means to maximize functional efficiency while minimizing lattice perturbation.^31,44^

Edge-doped MQDs typically exhibit enhanced chemical accessibility due to the lower coordination numbers and higher surface energy of edge atoms. Dopants introduced at these sites can interact directly with the surrounding environment, facilitating rapid and selective binding events. In contrast, basal-plane doping tends to induce more subtle electronic modulation, influencing long-range charge transport and overall electronic stability.^45,47^ The choice between edge and basal-plane doping thus reflects a strategic trade-off between localized reactivity and global electronic control.

Recent advances emphasize spatially resolved doping strategies that combine both approaches within a single MQD. By independently tuning edge and basal-plane dopant populations, researchers can decouple reactivity from stability, enabling multifunctional MQDs.^47,48^ This level of spatial control underscores the evolution of MQD design from compositional tuning to architectural engineering at the atomic scale.

### 2.5. Beyond nitrogen: expanding the dopant chemical space in MQDs

While nitrogen has been the most extensively explored dopant in MQDs, reliance on a single dopant limits the achievable property space. Expanding the dopant chemical palette to include sulfur, phosphorus, boron, and halogens introduces new dimensions of electronic and chemical control. Each heteroatom offers distinct electronegativity, size, and bonding characteristics, enabling tailored interactions that cannot be achieved through nitrogen doping alone. Multi-element and co-doping strategies further enrich this design space by introducing cooperative effects between different dopants. Such interactions can lead to emergent properties arising from dopant–dopant coupling, including enhanced charge polarization or selective site activation.^49,50^

Importantly, the reduced dimensionality of MQDs amplifies these cooperative effects, making them particularly sensitive to dopant combinations. Moving beyond nitrogen-centric designs aligns MQD research with broader trends in quantum materials and single-atom catalysis. By embracing chemical diversity and atomic precision, heteroatom-doped MQDs can be engineered as customizable platforms rather than fixed compositions. This shift is essential for unlocking their full potential in advanced functional systems.^51,52^

**Fig. 4:**
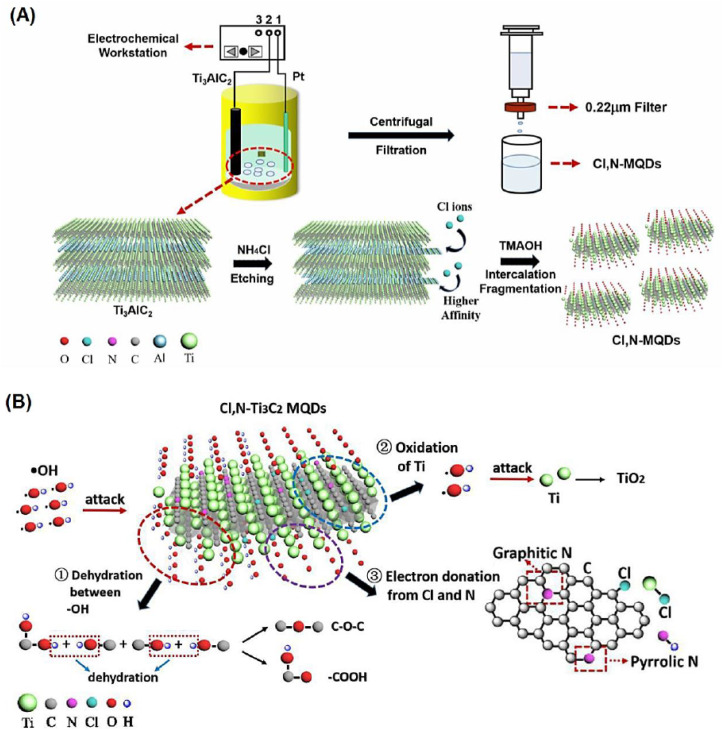
(A) Schematic illustration of the electrochemical fabrication of chlorine and nitrogen co-doped Ti_3_C_2_ MXene quantum dots with spatially differentiated dopant incorporation. (B) Conceptual representation of cooperative dopant-induced interfacial reactivity enabled by multi-element doping. This figure has been reproduced from ref. 52 with permission from Elsevier, copyright 2021.

Panel (A) in Fig. 4 schematically illustrates the controlled incorporation of chemically distinct dopants into Ti_3_C_2_ MQDs *via* an electrochemical etching strategy, highlighting the expansion of the dopant chemical space beyond nitrogen.^52^ In this system, nitrogen and chlorine are introduced through independent electrochemical pathways, selectively interacting with the carbon framework and titanium peripheries, respectively. Such spatially differentiated dopant incorporation demonstrates that MQDs can accommodate multiple heteroatoms with distinct electronegativity and bonding preferences within a single quantum-confined architecture. This co-doping strategy exemplifies how dopant diversity enables deliberate modulation of local electronic environments, moving MQD design beyond single-element doping toward chemically programmable systems.

Panel (B) in Fig. 4 conceptually depicts the cooperative action of Cl and N dopants in regulating interfacial reactivity, serving as an example of emergent behavior arising from dopant–dopant coupling. Rather than acting independently, the coexistence of dopants with contrasting electronic character generates complementary charge redistribution pathways, enhancing the interaction of MQDs with reactive species. From a materials design perspective, this illustrates how co-doping can produce functionalities that are not accessible through nitrogen doping alone, underscoring the importance of expanding the dopant palette in MQDs.

**Fig. 5:**
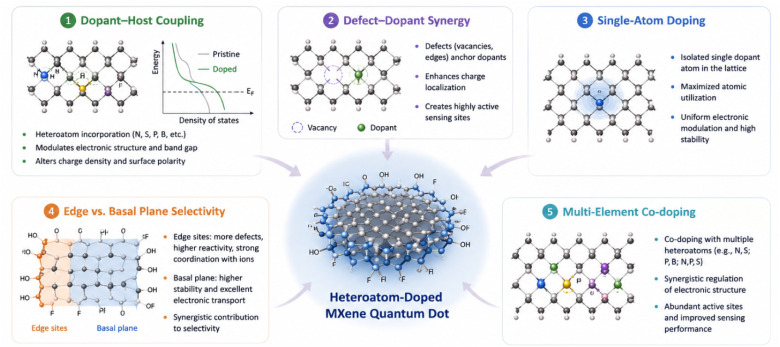
Schematic illustration of atomic-level engineering strategies in heteroatom-doped MQDs, highlighting dopant–host coupling, defect–dopant synergy, single-atom doping, edge *versus* basal-plane selectivity, and multi-element co-doping for tuning electronic structure, active sites, and selective transition-metal ion recognition.

Fig. 5 presents an integrated overview of the major atomic-level design strategies employed in heteroatom-doped MQDs. Unlike conventional compositional modification approaches, these strategies emphasize precise regulation of local electronic environments, defect distributions, and spatial dopant positioning within quantum-confined architectures. Dopant–host coupling and defect–dopant synergy collectively modulate charge redistribution and coordination behavior, while single-atom doping enables highly localized electronic control with minimal lattice disruption. In parallel, edge-selective and basal-plane doping introduce distinct reactivity and stability profiles, allowing decoupling of surface activity from global electronic transport. Multi-element co-doping further expands the accessible chemical space through cooperative electronic interactions between heteroatoms. Collectively, these atomic-level engineering approaches provide the structural and electronic foundation for enhanced selectivity, sensitivity, and multifunctionality in transition-metal ion sensing applications.

Table 1 provides a comparative overview of advanced atomic-level engineering strategies employed in heteroatom-doped MQDs. Rather than representing simple compositional modification, these strategies enable precise regulation of local electronic environments, defect distributions, and spatial dopant organization within quantum-confined architectures. Dopant–host coupling, defect-mediated electronic modulation, single-atom doping, and spatially selective edge/basal-plane engineering collectively govern charge redistribution, coordination behavior, and interfacial reactivity. In parallel, co-doping and chemical diversification strategies expand the accessible electronic and redox landscape of MQDs, enabling tailored recognition behavior toward transition-metal ions. Collectively, these approaches illustrate the evolution of MQD engineering from conventional bulk doping toward atomically programmable sensing architectures with tunable selectivity, stability, and multifunctionality.

**Table 1:** Advanced atomic-level engineering strategies in heteroatom-doped MQDs for selective transition-metal ion recognition

Design strategy	Dopant configuration	Spatial domain	Primary electronic effect	Structural/defect role	Functional outcome	Ref.
Dopant–host electronic coupling	N, S, P, B substitutional/interstitial doping	Edge + basal plane	Charge redistribution and orbital hybridization	Preserves lattice framework with localized perturbation	Tunable redox activity and selective ion coordination	30–33
Defect–dopant synergy	Vacancy-associated N/S doping	Vacancies and edge defects	Localized dipole formation and asymmetric charge density	Stabilization of defect-rich active sites	Enhanced affinity toward transition-metal ions	34–36
Single-atom doping	Isolated heteroatom incorporation	Edge or basal region	Discrete localized electronic states	Maintains structural integrity with minimal recombination	Ultra-selective and precision-controlled sensing	37–39
Low-concentration doping	Dilute N/S/P incorporation	Predominantly basal plane	Fine electronic band modulation	Reduced aggregation and defect-assisted stabilization	Improved photoluminescence stability/reactivity balance	40–43
Edge-selective doping	Edge-confined heteroatoms	MQD edge sites	High local charge density and surface reactivity	Exploits undercoordinated atomic environments	Rapid and accessible ion recognition	43–46
Basal-plane doping	Uniform basal-plane doping	Basal lattice region	Long-range electronic transport modulation	Minimal lattice distortion	Enhanced electronic stability and emission control	47–50
Multi-element Co-doping	N + S, N + P, B + S systems	Edge + basal combined	Synergistic charge polarization	Dopant–dopant electronic coupling	Multifunctional and enhanced sensing behavior	50–52
Heteroatom chemical expansion	Halogen, B, P, S incorporation	Edge/basal/interstitial	Electronegativity-driven orbital modulation	Cooperative structural stabilization	Expanded redox and analyte recognition capability	42–44
Spatially resolved doping	Segregated edge/basal doping	Architecturally differentiated MQDs	Decoupled reactivity and electronic transport	Selective defect utilization	Simultaneous stability and high chemical activity	49–51

### 2.6. Atomic structures of MQDs and transition-metal ions: structural basis for ion recognition

To further clarify the fundamental interactions underlying transition-metal ion sensing, it is important to consider the atomic structures of both MQDs and the target metal ions. MQDs are typically derived from layered MAX phases (M_*n*+1_AX_*n*_), where M represents an early transition metal (*e.g.*, Ti), A is an A-group element (such as Al), and X denotes carbon or nitrogen. After selective etching of the A layer, two-dimensional MXene sheets such as Ti_3_C_2_T_*x*_ or Ti_2_CT_*x*_ are produced, where T_*x*_ represents surface terminations including –O, –OH, and –F. When these sheets are further reduced to the quantum-dot scale, the resulting MQDs retain the hexagonal metal–carbon framework while exhibiting abundant edge sites and surface functional groups.

At the atomic level, the lattice structure consists of transition-metal layers sandwiched with carbon atoms, forming strong Ti–C bonds that provide structural stability and electronic conductivity. Surface terminations and edge defects introduce additional coordination sites capable of interacting with external species.^20–22^ These chemically active sites play a central role in ion recognition because they can coordinate with metal ions through electrostatic attraction, surface complexation, or electron-transfer processes.

Transition-metal ions such as Fe^3+^, Cu^2+^, Zn^2+^, and Co^2+^ possess partially filled d orbitals and well-defined coordination geometries. Their electronic configurations enable strong interactions with electron-rich surface groups on MQDs, particularly oxygen- or nitrogen-containing functional groups introduced through heteroatom doping. At the nanoscale interface, these ions may form coordination complexes with dopant atoms or surface ligands, leading to measurable changes in fluorescence, charge transfer, or electrochemical response.^13–18^

Therefore, understanding the atomic structures of both MQDs and transition-metal ions provides essential insight into the mechanisms of selective ion recognition. The interplay between the quantum-confined MXene lattice, surface functional groups, and the coordination chemistry of transition-metal ions ultimately governs sensing sensitivity and selectivity in heteroatom-doped MQD systems.

## 3. Emerging recognition paradigms in MQDs: from static fluorescence signals to intelligent sensing systems

### 3.1. From intensity-based readouts to information-rich ratiometric recognition architectures

Conventional fluorescence-based sensing strategies have historically relied on single-channel intensity changes as the primary analytical signal. While straightforward, such approaches are inherently vulnerable to environmental fluctuations, probe concentration variations, and instrumental instability. In response, ratiometric recognition architectures have emerged as a transformative paradigm for MQD-based sensing systems. These architectures generate two or more correlated optical signals, enabling internal calibration and enhanced analytical robustness.^53–55^

Ratiometric systems encode recognition events as signal ratios rather than absolute intensities, significantly improving accuracy in complex matrices. In the context of MQDs, this paradigm can be implemented through dual-emission systems, energy transfer cascades, or coupling MQDs with secondary emissive species. Importantly, the ratiometric concept is not merely a signal-processing upgrade but a fundamental shift in how recognition information is represented and interpreted. By converting chemical interactions into multidimensional optical outputs, these systems increase information density per sensing event.^56,57^

Beyond analytical reliability, ratiometric architectures enable discrimination between closely related analytes by exploiting differential modulation of multiple channels. This capability is particularly relevant for transition-metal ions, which often exhibit overlapping coordination behavior. By designing recognition systems that respond along orthogonal optical axes, MQD-based platforms can transcend the selectivity limitations of single-signal probes. At a systems level, ratiometric recognition aligns MQD sensing with broader trends in analytical chemistry toward self-referenced and error-tolerant measurement strategies.^59–61^ As sensing environments become increasingly complex—ranging from environmental samples to biological fluids—the transition from intensity-based to information-rich readouts represents a critical evolutionary step for MQD-enabled recognition technologies.

**Fig. 6:**
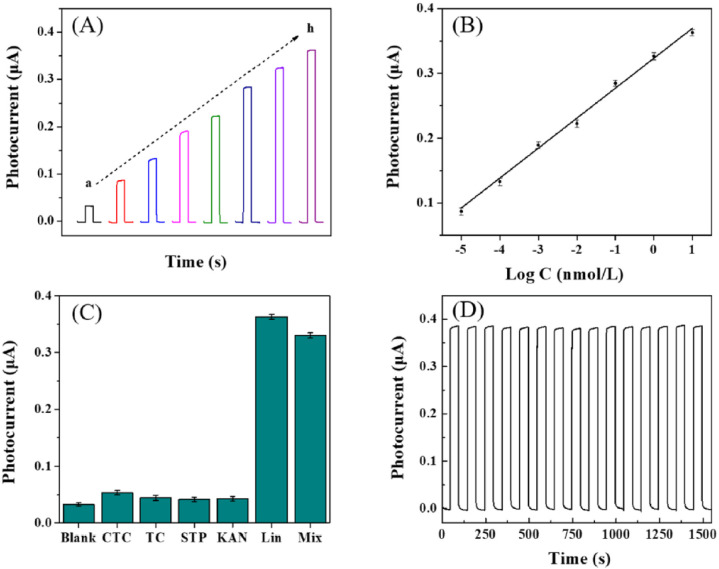
Analytical performance of the PEC aptasensor. (A) Photocurrent responses at different Lin concentrations. (B) Calibration curve of photocurrent *versus* logarithmic concentration of Lin. (C) Selectivity evaluation against interfering antibiotics. (D) Stability test under repeated illumination cycles. This figure has been reproduced from ref. 60 with permission from Elsevier, copyright 2022.

The panels in Fig. 6 illustrate the analytical performance of the MQD-based photoelectrochemical (PEC) aptasensor and highlight how signal modulation can encode recognition events with high reliability. Panel (A) shows the photocurrent responses obtained at different concentrations of lincomycin (Lin), where the gradual increase in signal reflects the sensitive interaction between the target molecule and the MQD-based sensing interface. This concentration-dependent modulation demonstrates how chemical recognition can be translated into measurable electronic outputs. Panel (B) presents the corresponding calibration relationship between photocurrent and the logarithm of Lin concentration, revealing a wide linear detection range and an ultralow detection limit, which indicates the high sensitivity of the sensing platform. Meanwhile, panel (C) evaluates the selectivity of the system by comparing the response to Lin with several structurally related antibiotics, confirming that the sensor maintains a highly specific response even in the presence of potential interferents. Finally, panel (D) demonstrates the operational stability of the aptasensor under repeated light on/off cycles, showing negligible signal degradation. Collectively, these results illustrate how MQD-integrated sensing architectures convert molecular recognition into reproducible and information-rich signal outputs, a key requirement for the development of robust and advanced recognition systems.

### 3.2. Adaptive and stimuli-responsive recognition systems: toward dynamic sensing behavior

Traditional sensing platforms are typically static, designed to respond once to a specific analyte under fixed conditions. In contrast, adaptive and stimuli-responsive recognition systems introduce dynamic behavior into MQD-based sensing, allowing the system to adjust its response based on external or internal stimuli. This paradigm draws inspiration from biological recognition processes, where sensing is inherently context-dependent and reversible. Adaptive recognition systems can modulate their sensing performance in response to factors such as pH, ionic strength, competing species, or sequential analyte exposure.^62–64^ Rather than being sources of interference, these variables become integrated control parameters that shape the sensing outcome. In MQD-based platforms, such adaptability enables multi-stage recognition processes, where one stimulus activates or deactivates sensitivity toward another target.

Stimuli-responsive behavior also enables reversible and reusable sensing systems. By designing recognition architectures that can switch between “on,” “off,” or intermediate states, MQD-based probes can perform multiple sensing cycles without structural degradation. This dynamic capability is particularly attractive for real-time monitoring applications, where continuous or repeated measurements are required. At a conceptual level, adaptive recognition represents a departure from single-analyte detection toward system-level responsiveness. MQDs serve not only as signal transducers but as integral components of responsive networks that process environmental information.^65,66^ This shift elevates MQD sensing from a passive measurement tool to an active analytical system capable of decision-making based on changing conditions.

### 3.3. Multimodal recognition strategies: integrating optical, electrochemical, and visual outputs

As sensing challenges grow more complex, reliance on a single transduction mode is increasingly insufficient. Multimodal recognition strategies address this limitation by integrating multiple signal outputs—such as optical, electrochemical, and visual responses—within a single MQD-based platform. These systems leverage complementary detection modalities to enhance confidence, versatility, and user accessibility. In multimodal architectures, MQDs often act as a central recognition element capable of interfacing with diverse signal pathways. Optical signals may provide high sensitivity, while electrochemical responses offer quantitative precision and compatibility with portable devices. Visual outputs, such as color changes or test-strip readouts, enable rapid and instrument-free detection.^67,68^ The convergence of these modes creates layered information channels that reinforce analytical conclusions.

Importantly, multimodal recognition does not simply replicate the same information across different outputs. Instead, each modality can be tailored to respond to distinct aspects of the recognition event, such as concentration range, kinetics, or competing species. This functional differentiation enhances the analytical depth of MQD-based systems and reduces ambiguity in real-world applications. From a translational perspective, multimodal strategies bridge the gap between laboratory-scale performance and field deployment. By accommodating both high-end instrumentation and low-resource settings, MQD-based multimodal sensors align with global trends toward decentralized and user-friendly analytical technologies. This versatility positions MQDs as adaptable platforms rather than single-purpose probes.^69–71^

**Fig. 7:**
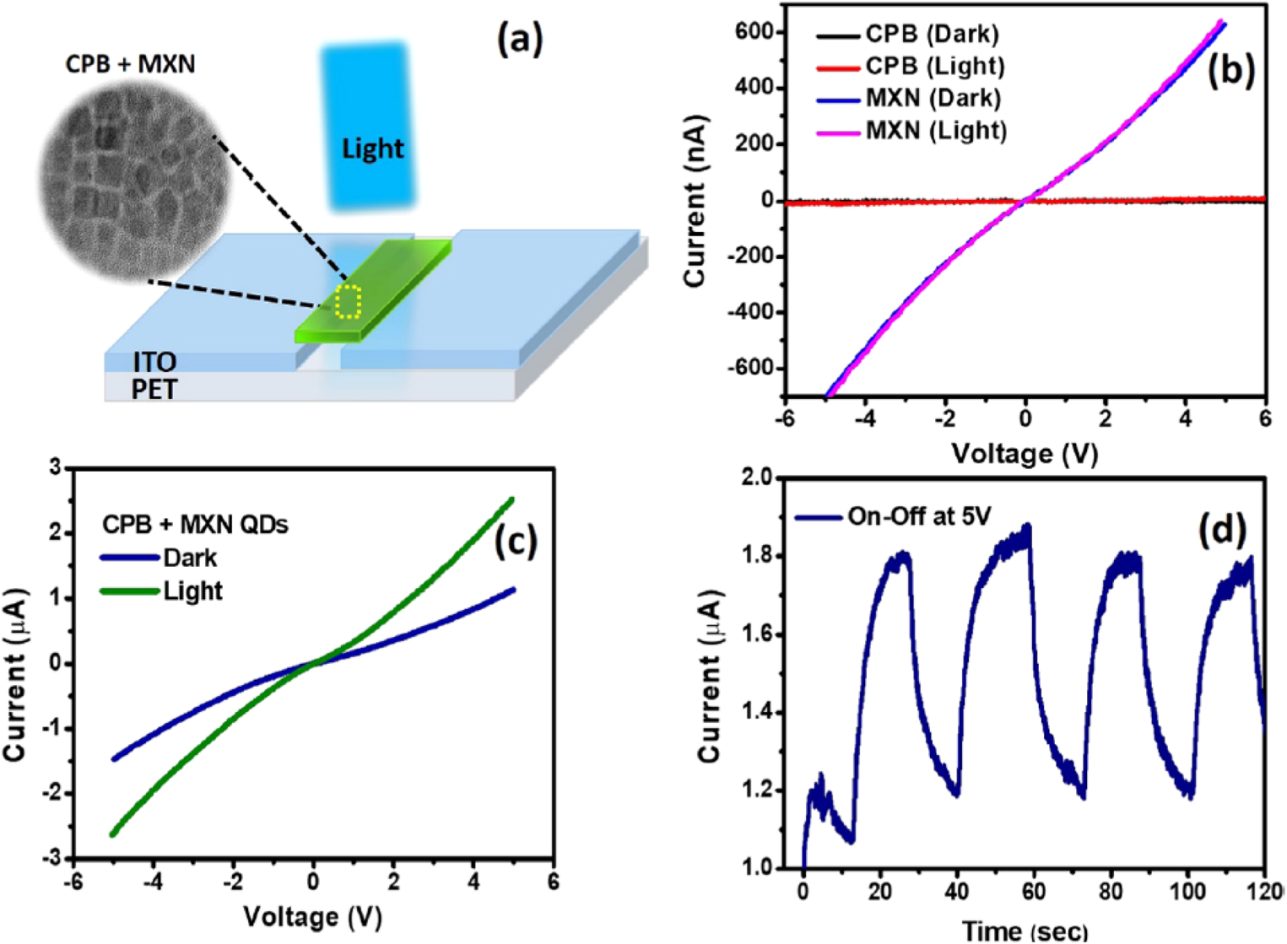
(a) Device architecture of the CPB–MXN QD photodetector; (b) *I*–*V* characteristics of CPB and MXN QD films in dark and under illumination; (c) enhanced photocurrent response of the CPB–MXN composite device; (d) time-dependent photocurrent switching during repeated light on/off cycles. This figure has been reproduced from ref. 71 with permission from American Chemical Society, copyright 2020.

Fig. 7 illustrates how the CPB–MXN quantum-dot composite operates as a platform capable of integrating optical excitation with electrical signal readout, highlighting the principles of multimodal recognition architectures. Panel (a) presents the device configuration used to probe the photoelectrical behavior of the QD films. The current–voltage curves in panel (b) show that individual CPB and MXN QD films exhibit weak electrical conductivity and limited photocurrent under illumination, indicating inefficient carrier transport and rapid recombination within isolated nanocrystals. In contrast, the CPB–MXN composite device in panel (c) displays markedly enhanced conductivity and a pronounced photocurrent response when illuminated. This behavior suggests efficient interfacial charge separation and transfer between CPB and MXN domains, which creates a conductive pathway that converts optical excitation into measurable electrical output. The dynamic photocurrent switching observed in panel (d) during repeated illumination cycles further confirms the reversible and stable photoresponse of the composite system. Together, these observations demonstrate how MQD-based hybrid structures can couple optical stimulation with electrical transduction, providing complementary signal channels that enhance detection reliability and analytical versatility. Such integration of photonic and electronic responses exemplifies the operational principle of multimodal sensing platforms, where different output modalities reinforce the interpretation of the same recognition event.

### 3.4. Logic-gated and sequential recognition systems: encoding chemical events as logical operations

Logic-gated recognition systems represent a conceptual leap in chemical sensing, transforming MQD-based platforms into information-processing devices. In these systems, chemical inputs—such as the presence of specific ions or stimuli—are treated as logical variables that produce defined outputs based on Boolean or multi-valued logic rules. This approach enables highly selective recognition through combinatorial decision-making rather than single-response thresholds. Sequential recognition architectures further extend this concept by requiring ordered input events to generate a measurable output. Such systems can discriminate analytes not only by identity but also by interaction sequence, offering a powerful means of suppressing false positives. MQDs are particularly well-suited for these architectures due to their fast response times and compatibility with multiple signal modulation mechanisms.^58,66^

Logic-gated recognition is especially valuable in environments containing multiple potential interferents. By encoding selectivity into logical conditions rather than relying solely on chemical affinity, MQD-based systems achieve higher specificity under realistic conditions. This strategy mirrors computational principles, effectively embedding basic information processing within the sensing material itself. At a broader level, logic-based recognition reflects the convergence of sensing and computation. MQD platforms capable of executing logical operations blur the boundary between analytical chemistry and molecular information science, opening pathways toward intelligent sensing systems that respond selectively based on predefined decision rules.^42,72^

**Fig. 8:**
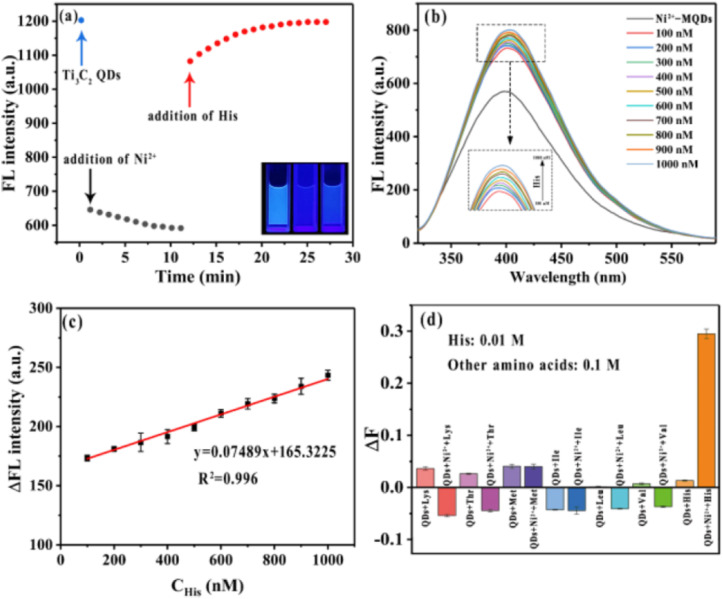
Sequential fluorescence recognition of histidine using MQDs. (a) Time-dependent fluorescence response after sequential addition of Ni^2+^ and His. (b) Fluorescence recovery of Ni^2+^–MQDs at different His concentrations. (c) Calibration curve of fluorescence recovery *versus* His concentration. (d) Selectivity of the sensing system toward His over other amino acids. This figure has been reproduced from ref. 72 with permission from American Chemical Society, copyright 2021.

Fig. 8 illustrates a sequential recognition process in which MQDs operate as a fluorescence transduction platform responding to two chemical inputs in a logic-like manner. As shown in panel (a), the FL intensity of MQDs is rapidly quenched upon the introduction of Ni^2+^, indicating the formation of a coordination interaction that suppresses emission. When histidine (His) is subsequently introduced, the fluorescence signal recovers due to the stronger complexation between Ni^2+^ and His, which disrupts the Ni^2+^–MQD interaction. This stepwise signal modulation demonstrates a sequential sensing mechanism in which the final output depends on the ordered introduction of two chemical species. Panel (b) further shows that the fluorescence recovery increases progressively with raising His concentration, reflecting the quantitative responsiveness of the system. The corresponding calibration curve in panel (c) confirms a linear relationship between fluorescence recovery and His concentration within the tested range, highlighting the analytical capability of the recognition architecture. Finally, panel (d) evaluates the selectivity of the system against other essential amino acids, demonstrating that significant fluorescence recovery occurs only in the presence of His. Together, these results exemplify how MQD-based sensing platforms can encode chemical events as ordered signal transformations, consistent with the principles of sequential and logic-gated recognition systems.

### 3.5. Toward intelligent and data-integrated recognition platforms using MQDs

The future of MQD-based recognition lies in the integration of sensing systems with data analytics and intelligent decision frameworks. Rather than treating recognition as an isolated measurement, emerging platforms aim to contextualize MQD signals within broader data streams, enabling pattern recognition, anomaly detection, and predictive analysis. Intelligent recognition systems can leverage temporal signal evolution, multivariate outputs, and historical data to enhance reliability and interpretability. MQDs, with their tunable and multiplexed response capabilities, provide rich datasets well-suited for algorithmic analysis. This synergy enables the transition from threshold-based detection to data-driven recognition models.^73–75^

Such integration is particularly relevant for continuous monitoring applications, where large volumes of data must be processed in real time. By coupling MQD-based sensors with computational tools, sensing platforms can adapt detection strategies dynamically, identify trends, and issue actionable outputs rather than raw signals. Ultimately, the evolution toward intelligent recognition systems redefines the role of MQDs in sensing technologies. They become components of adaptive analytical ecosystems rather than standalone probes. This paradigm shift aligns MQD research with broader movements toward smart materials and autonomous sensing infrastructures.^75,76^

Table 2 summarizes the evolution of MQD-based recognition systems from conventional single-channel fluorescence probes toward adaptive, multimodal, and data-integrated sensing architectures. Traditional intensity-based sensing provides operational simplicity but remains highly susceptible to environmental fluctuations and matrix-dependent interference. Emerging paradigms, including ratiometric, stimuli-responsive, multimodal, logic-gated, and sequential recognition systems, overcome these limitations by introducing self-calibration, dynamic signal modulation, and multidimensional analytical outputs. In parallel, intelligent data-integrated platforms combine MQD-generated signals with computational processing, enabling predictive analysis and autonomous sensing behavior. Collectively, these developments demonstrate the transformation of MQDs from passive fluorescence transducers into programmable and information-rich sensing systems capable of selective, adaptive, and real-time chemical recognition under complex operating conditions.

**Table 2:** Emerging recognition architectures and intelligent sensing paradigms in MQD-based platforms

Recognition strategy	Signal output	Recognition mechanism	Dynamic capability	Major analytical advantage	Application relevance	Ref.
Intensity-based fluorescence	Single-emission fluorescence	Direct quenching or enhancement	Static response	Simple and rapid signal generation	Conventional laboratory ion sensing	53–55
Ratiometric recognition	Dual-emission fluorescence	Ratio-based self-calibrated signaling	Internal referencing	Reduced environmental artifacts and enhanced quantification	Complex matrices and ion discrimination	56–58
Stimuli-responsive systems	Modulated fluorescence response	pH/ionic-strength/sequential control	Reversible adaptive sensing	Tunable sensitivity and reusable operation	Dynamic environmental monitoring	59–61
Multimodal recognition	Optical/electrochemical/visual outputs	Integrated multi-signal transduction	Parallel signal generation	Increased analytical confidence and reduced false positives	Portable and field-deployable sensing	62–64
Logic-gated recognition	Fluorescence/ratiometric outputs	Boolean or multi-input processing	Sequential input dependency	High specificity under interferent-rich conditions	Intelligent chemical recognition	65–67
Sequential recognition	Multi-step fluorescence modulation	Ordered analyte interaction pathways	Time-dependent response evolution	Improved discrimination fidelity	Multi-ion and complex sample analysis	68–70
Intelligent data-integrated platforms	Multivariate sensing signals	Algorithm-assisted signal interpretation	Real-time adaptive analytics	Predictive recognition and pattern analysis	Autonomous monitoring systems	71–73
FRET-based recognition systems	Energy-transfer fluorescence	MQD donor–acceptor interaction	Stimuli-dependent energy transfer	Multiplexed and orthogonal sensing capability	Bioimaging and multiplex detection	74–76

## 4. Transition-metal ion recognition using heteroatom-doped MQDs: performance metrics and application-oriented analysis

Although a wide range of sensing platforms—including carbon quantum dots, graphene-based materials, organic fluorophores, and metal–organic frameworks—have been explored for detecting transition-metal ions such as Fe^3+^, Cu^2+^, Zn^2+^, and Mn^2+^, the present review specifically focuses on MQD-based sensing systems due to their unique optical properties and tunable surface chemistry.

### 4.1. High-sensitivity recognition of Fe^3+^ ions: redox-active MQD platforms for environmental monitoring

Iron(iii) ion detection remains one of the most mature and analytically demanding application areas for MQD-based fluorescent sensors, owing to the strong redox activity of Fe^3+^ and its widespread environmental relevance. MQD platforms functionalized with heteroatom-containing surface groups demonstrate a remarkable ability to convert Fe^3+^–surface interactions into measurable fluorescence responses across broad concentration ranges, enabling both ultra-trace detection and routine environmental monitoring.^77,78^

In amino-functionalized Ti_3_C_2_T_*x*_ MQDs, Fe^3+^ recognition is dominated by redox interactions between the highly oxidizing Fe^3+^ ions and the electron-rich MQD surface. These interactions lead to efficient fluorescence quenching, producing a highly sensitive analytical signal with linear behavior spanning nanomolar to micromolar concentrations.^77^ Detection limits in the low nanomolar regime position such platforms well below regulatory thresholds for drinking water, making them particularly suitable for early-warning monitoring of iron contamination. The wide dynamic range further supports their applicability across diverse environmental conditions, from pristine water sources to industrial effluents.

Alternative MQD-based Fe^3+^ sensing strategies rely on electrostatic and aggregation-induced quenching effects rather than direct redox coupling. MQDs prepared through solvent-regulated ultrasonic routes exhibit rapid fluorescence suppression upon Fe^3+^ exposure, attributed to ion-induced aggregation processes that disrupt emissive pathways.^78^ Although these systems typically operate with micromolar detection limits, their rapid response kinetics and straightforward preparation protocols offer distinct practical advantages. Such characteristics are especially valuable for screening-level analyses or on-site testing scenarios where operational simplicity and speed are prioritized over extreme sensitivity. Taken together, these complementary approaches illustrate the adaptability of heteroatom-doped MQDs for Fe^3+^ monitoring. Redox-active platforms emphasize sensitivity and trace-level detection, while aggregation-driven systems favor robustness and ease of deployment.^15,17^ This performance diversity highlights how MQD-based Fe^3+^ sensors can be strategically selected or engineered to meet specific analytical objectives across varied environmental monitoring contexts.

### 4.2. Cu^2+^ ion detection *via* fluorescence quenching and ratiometric amplification

In this subsection, MQD-based sensing strategies for the detection of Cu^2+^ ions are discussed. Copper(ii) ion detection represents a critical analytical task due to the ion's catalytic activity, biological relevance, and frequent presence in natural and industrial water systems. Heteroatom-doped MQD platforms demonstrate that Cu^2+^ can be sensitively detected through both direct fluorescence intensity modulation and more advanced ratiometric signal amplification strategies, enabling flexible adaptation to different analytical requirements.^79,80^ Amino-rich, covalently nitrogen-doped MQDs exhibit pronounced fluorescence quenching upon Cu^2+^ coordination, providing a simple yet effective sensing modality.^79^ This response remains linear over a wide concentration window extending from submicromolar to several hundred micromolar levels, covering concentration ranges relevant to environmental regulation and industrial discharge monitoring. Detection limits well below guideline values ensure adequate sensitivity, while the incorporation of masking strategies enables reliable discrimination against competing metal ions in complex aqueous matrices. The demonstrated compatibility with real water samples confirms the operational relevance of this intensity-based sensing approach.

**Fig. 9:**
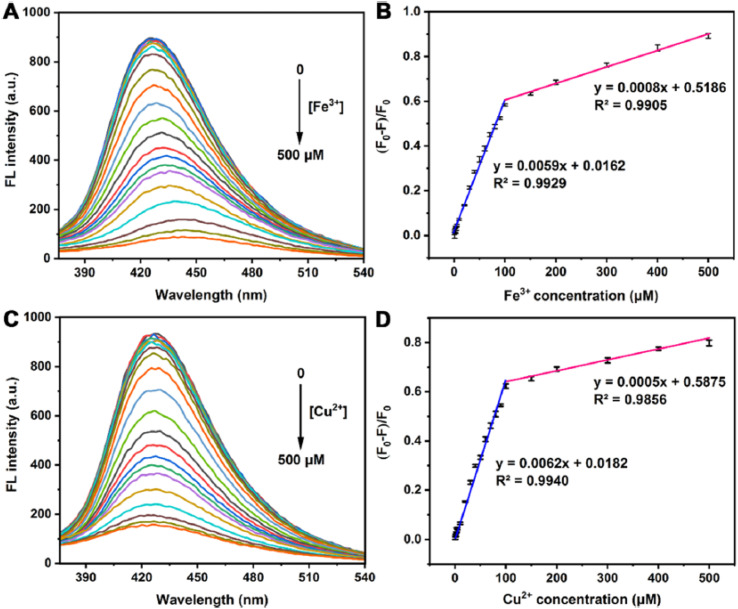
Fluorescence sensing performance of N-doped MQDs toward Fe^3+^ and Cu^2+^ ions. (A and C) Fluorescence emission spectra of N-MQDs recorded at 330 nm in the presence of increasing concentrations (0–500 µM) Cu^2+^ ion, respectively. (B and D) Linear relationship between normalized fluorescence quenching efficiency ((*F*_0_ − *F*)/*F*_0_) and metal ion concentration, demonstrating sensitive and quantitative detection in the low-concentration range. This figure has been reproduced from ref. 79 with permission from American Chemical Society, copyright 2022.

As illustrated in Fig. 9A and C, the fluorescence emission of N-MQDs shows quenching behavior in the presence of Cu^2+^ ions; the same sensing platform was also reported to respond to Fe^3+^, indicating its multi-ion detection capability. With increasing metal ion concentration from 0.5 to 500 µM, the emission intensity decreases monotonically, indicating efficient interaction between the paramagnetic metal ions and the emissive states of the MQDs. In the case of Cu^2+^, this strong quenching response is attributed to the formation of stable coordination complexes between Cu^2+^ ions and amino functionalities introduced through nitrogen doping.^60,61^ Such coordination facilitates non-radiative electron or energy transfer processes, effectively depleting the excited-state population and suppressing fluorescence emission, consistent with a charge-transfer-assisted quenching mechanism.

Quantitative analysis of the sensing performance, shown in Fig. 9B and D, reveals a robust linear relationship between the normalized fluorescence quenching efficiency ((*F*_0_ − *F*)/*F*_0_) and Cu^2+^ concentration over the 0.5–100 µM range. The low detection limit of 0.15 µM underscores the high sensitivity achieved through heteroatom doping, which enhances both the intrinsic fluorescence intensity of the MQDs and their affinity toward Cu^2+^ ions. Compared with undoped MQDs, the aminated N-MQDs display significantly amplified quenching responses, highlighting the critical role of surface electronic structure and functional group engineering in regulating metal ion–MQD interactions. These results align well with the broader framework of fluorescence modulation and signal amplification, demonstrating how tailored MQD platforms enable reliable, selective, and ultrasensitive Cu^2+^ detection in aqueous environments.

In contrast, ratiometric MQD-based Cu^2+^ sensors encode ion recognition as a ratio between two fluorescence emissions, significantly enhancing analytical robustness.^80^ By decoupling the analytical signal from absolute intensity values, ratiometric amplification minimizes the influence of external variables such as excitation fluctuations, probe concentration variations, and photobleaching. As a result, detection limits are pushed into the nanomolar range, representing a substantial improvement in sensitivity and reliability compared to single-channel fluorescence probes.

From a performance perspective, intensity-based quenching sensors offer simplicity, broad dynamic ranges, and ease of implementation, making them suitable for routine monitoring applications. Ratiometric systems, while more complex, provide superior sensitivity and measurement stability, particularly in challenging or variable sample environments. Together, these complementary strategies establish heteroatom-doped MQDs as versatile and competitive platforms for Cu^2+^ detection across laboratory and real-world analytical scenarios.

### 4.3. Zn^2+^ recognition through fluorescence enhancement and sequential signal modulation

Zinc(ii) ion detection presents a unique challenge in transition-metal sensing due to its fully filled d-orbitals and minimal participation in redox processes. Conventional fluorescence quenching strategies, which rely on electron-transfer or redox interactions, often produce weak or non-selective signals when applied to Zn^2+^. Nitrogen-doped-MQDs overcome this limitation by employing fluorescence enhancement as the primary transduction mechanism, converting subtle coordination-induced electronic perturbations into strong emissive outputs.

In this system, Zn^2+^ coordination induces a pronounced fluorescence “turn-on” response, with linear detection behavior in the low micromolar range. The enhancement mechanism stems from stabilization of the excited-state electronic structure of the MQDs, promoting radiative recombination and suppressing non-radiative decay. This approach contrasts with conventional quenching-based sensors and allows Zn^2+^ to be detected with minimal interference from quenching-prone transition metals. The resulting analytical signal is highly sensitive, with low detection limits and good reproducibility across repeated measurements, demonstrating the robustness of the fluorescence enhancement strategy.^81^

A notable advancement of this platform is its sequential “off–on–off” modulation capability. After initial Zn^2+^-induced fluorescence enhancement, secondary analytes can modulate the emission reversibly, enabling multi-step sensing applications. This feature allows selective secondary detection without compromising Zn^2+^ quantification, which is particularly advantageous in complex sample matrices such as environmental waters and food extracts. By exploiting the reversible nature of coordination interactions, the platform integrates both primary detection and functional adaptability.^17,61^

The system's practical utility has been validated in real-world matrices, highlighting the potential of fluorescence enhancement-based MQDs for operationally relevant Zn^2+^ sensing. Compared to redox- or quenching-dominated platforms, this strategy provides superior selectivity in mixed-metal environments, where non-redox ions would otherwise fail to produce measurable or distinguishable signals. Overall, the approach illustrates how diversifying signal transduction modes broadens the functional capabilities of MQD-based sensors, establishing a pathway for analytically challenging ions to be detected with high fidelity.

### 4.4. Mn^2+^ detection using ratiometric dual-emission MQD-based probes

Manganese(ii) detection poses a particular analytical challenge due to the weak perturbation it induces in fluorescence-based systems. As Mn^2+^ is neither strongly redox-active nor a potent quencher, single-emission intensity measurements are prone to signal drift, matrix effects, and limited sensitivity. Ratiometric dual-emission MQD platforms address these limitations by generating an internal reference signal alongside the Mn^2+^-responsive emission, producing a self-calibrating, highly robust analytical output. In this configuration, Mn^2+^ coordination triggers a new emission band, while a separate, metal-insensitive emission serves as a reference. The ratio of these two intensities provides a linear and concentration-dependent signal across the low micromolar range, with detection limits extending into the nanomolar regime. This self-normalizing behavior enhances quantitative reliability and mitigates interference from environmental variability, probe concentration fluctuations, and excitation instability, which are common limitations of single-channel sensors.^82^

The ratiometric approach also confers superior selectivity in complex ionic matrices, where competing divalent cations or varying ionic strength might otherwise compromise detection. Testing in commercially available mineral waters confirmed both high selectivity and reproducibility. Importantly, the platform can be translated into visual and portable formats, such as test strips, enabling rapid on-site analysis without the need for sophisticated instrumentation. This combination of sensitivity, selectivity, and deployability addresses the growing demand for practical water quality monitoring tools. From a design perspective, ratiometric MQDs exemplify how heteroatom doping and engineered emission properties can overcome intrinsic sensing limitations of chemically inert or weakly interacting metal ions. By leveraging dual-emission behavior and internal referencing, these systems maintain analytical fidelity while broadening applicability beyond laboratory conditions.^53,60^ The Mn^2+^ platform thus establishes a generalizable framework for incorporating ratiometric signal strategies into next-generation MQD-based metal ion sensors.

**Fig. 10:**
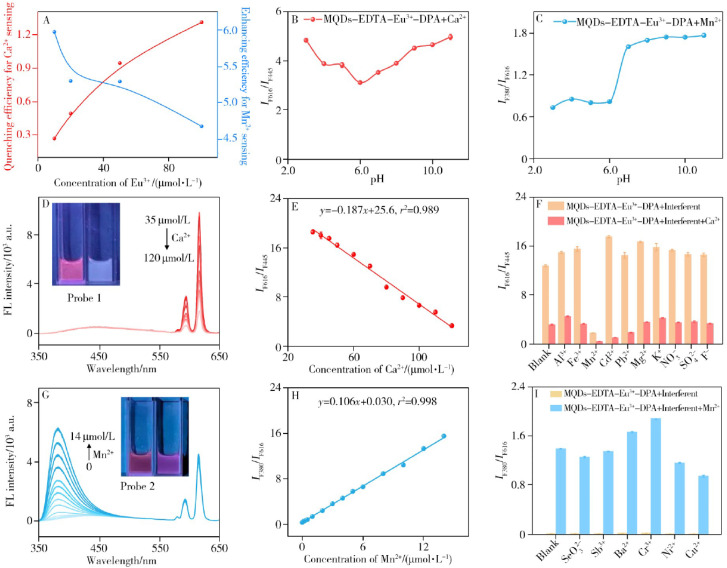
Ratiometric fluorescence sensing of Ca^2+^ and Mn^2+^ using the MQDs–EDTA–Eu^3+^–DPA probe. (A) Effect of Eu^3+^ concentration on detection sensitivity. (B and C) Influence of pH on Ca^2+^ and Mn^2+^ sensing performance. (D) Dual-emission fluorescence spectra and (E) corresponding ratiometric calibration curve (*I*_F616_/*I*_F445_) for Ca^2+^ detection. (F) Selectivity evaluation in drinking water matrices. (G) Fluorescence spectra and (H) ratiometric calibration curve (*I*_F380_/*I*_F616_) for Mn^2+^ detection. (I) Selectivity evaluation in natural mineral water matrices. Insets: fluorescence photographs before and after addition of Mn^2+^. This figure has been reproduced from ref. 82 with permission from Fxcsxb, copyright 2025.

Fig. 10 systematically illustrates the design rationale and analytical robustness of the dual-emission ratiometric MQD-based probe (MQDs–EDTA–Eu^3+^–DPA) for Mn^2+^ detection. Optimization of experimental parameters reveals that both Eu^3+^ concentration and solution pH critically govern sensing performance. As shown in Fig. 10A, the ratiometric response toward Mn^2+^ is maximized at a Eu^3+^ concentration of 100 µM, where balanced emission intensities enable optimal signal contrast. Meanwhile, Fig. 10B and C demonstrate that near-neutral pH conditions favor stable and reproducible detection of Mn^2+^ ion, justifying the use of PBS buffer at pH 7.0 for subsequent measurements. This optimization step is essential for ensuring that ratiometric outputs reflect genuine metal–probe interactions rather than environmental fluctuations.

Under UV excitation at 265 nm, the probe exhibits two well-resolved emission bands: a blue emission at 445 nm originating from the MQDs and a red emission at 616 nm associated with the Eu^3+^–DPA complex (Fig. 10D). Upon addition of Ca^2+^ ions, the red Eu^3+^-centered emission is selectively attenuated while the MQD emission remains invariant, yielding a visually discernible color transition from red to light blue (inset of Fig. 10D). The resulting linear relationship between *I*_F616_/*I*_F445_ and Ca^2+^ concentration (Fig. 10E) confirms the role of the MQD emission as an internal reference channel, highlighting the self-calibrating nature of the ratiometric strategy.

In contrast, Mn^2+^ detection proceeds through a distinct dual-modulation pathway. As shown in Fig. 10G, coordination between Mn^2+^ ions and DPA ligands induces the emergence of a new purple emission band at 380 nm, attributed to the Mn^2+^–DPA complex, while the Eu^3+^-based emission at 616 nm is gradually suppressed. This dual-emission evolution produces a clear color change from red to purple (inset of Fig. 9G) and enables construction of a highly linear ratiometric calibration curve based on *I*_F380_/*I*_F616_. The low detection limit in the nanomolar regime underscores the effectiveness of ratiometric amplification in overcoming the inherently weak fluorescence perturbation typically associated with Mn^2+^ ions.

Selectivity studies further validate the analytical reliability of this platform. As depicted in Fig. 10F and I, common coexisting ions defined in drinking water and natural mineral water standards induce negligible interference in the ratiometric response. This resistance to matrix effects, combined with internal referencing and visually distinguishable fluorescence changes, exemplifies how dual-emission MQD architectures can deliver high-fidelity Mn^2+^ sensing under realistic conditions.

### 4.5. Composition-engineered MQDs: structure–property correlations in Nb_2_C-based systems

The development of Nb_2_C-based MQDs introduces critical material diversity in the MQD field, which has traditionally focused on Ti_3_C_2_-derived systems. Sulfur and nitrogen co-doping of Nb_2_C MQDs yields bright green fluorescence, stable photophysical characteristics, and robust coordination behavior toward Cu^2+^ ions. This material choice demonstrates that high-performance transition-metal ion sensing is achievable beyond Ti-based MQDs and highlights the role of MXene composition in tuning electronic structure and surface chemistry. Cu^2+^ detection using Nb_2_C MQDs occurs in the low micromolar range, offering sufficient sensitivity for biological and environmental applications. While the detection limits may not reach the extreme nanomolar values reported for some Ti-based systems, the Nb_2_C MQDs provide complementary advantages: excellent photostability and biocompatibility.^83^ The sustained fluorescence under prolonged excitation allows integration into bioimaging workflows, enabling dual functionality in ion sensing and cellular imaging.

The platform exemplifies a performance balance that extends MQD applicability beyond ultra-trace detection. By engineering MXene composition and heteroatom dopants, the sensing system achieves a combination of moderate sensitivity, functional stability, and application versatility. This approach underscores the importance of tailoring MQD materials to meet multi-dimensional requirements, such as compatibility with biological environments, optical robustness, and simultaneous analytical and imaging capabilities.^40,53^ Overall, Nb_2_C MQDs highlight a strategic route for expanding MQD-based sensors to alternative transition-metal targets, demonstrating that material diversity can unlock multifunctional sensing performance. These findings reinforce the broader potential of heteroatom-doped MQDs to serve as flexible, tunable platforms for selective transition-metal ion recognition, bridging environmental and bioanalytical applications.

As shown in Table 3, the differences in analytical performance among heteroatom-doped MQD-based sensors, particularly in terms of detection limit, are closely related to their structural design, surface chemistry, and signal-output mode. Sensors exhibiting nanomolar-level LODs generally benefit from stronger and more specific interactions between the target metal ions and the doped MQD surface. In this context, heteroatom doping creates defect-rich active sites and modulates the electronic structure of MQDs, which enhances charge transfer and fluorescence responsiveness upon metal-ion binding. At the same time, surface functional groups such as amino, hydroxyl, or sulfur-containing moieties improve coordination affinity and contribute to higher selectivity. Table 3 also indicates that sensing platforms based on FRET or ratiometric fluorescence often achieve superior sensitivity compared with conventional single-intensity quenching systems, because they reduce background interference and improve signal precision. Therefore, lower detection limits are not determined by a single factor, but rather by the synergistic combination of dopant engineering, interfacial recognition chemistry, and advanced signal transduction strategies.

**Table 3:** Quantitative performance metrics of heteroatom-doped MQD sensors for transition-metal ion detection

MQDs type/modification	Target analyte	Detection strategy	Linear range	LOD	Key notes	Ref.
Amino-functionalized Ti_3_C_2_T_*x*_ MQDs	Fe^3+^	Fluorescence sensing	nM to µM range	2 nM	Ultrasensitive detection *via* redox interaction between Fe^3+^ and Ti_3_C_2_T_*x*_ MQDs	77
MXene QDs regulated by DMF	Fe^3+^	Aggregation-induced fluorescence quenching	Not specified	1.4 µM	Sensitivity: 0.6377 mM^−1^	78
Covalently N-doped Ti_3_C_2_ MQDs	Fe^3+^/Cu^2+^	Fluorescence sensing	0.5–500 µM	0.17 µM (Fe^3+^); 0.15 µM (Cu^2+^)	Amino-rich surface improves sensitivity and stability	79
N-doped Ti_3_C_2_ MQDs	Cu^2+^/d-penicillamine	FRET-based ratiometric fluorescence	Not specified	3.0 nM (Cu^2+^); 0.115 µM (D-PA)	Ratiometric fluorescence based on ox-OPD formation	80
N-doped MQDs	Zn^2+^/oxalic acid	“Off–On–Off” fluorescence sensing	0–20 µM	0.127 µM (Zn^2+^); 0.883 µM (OA)	ICT mechanism with Zn^2+^ and coordination with OA	81
MQDs–EDTA–Eu^3+^–DPA probe	Ca^2+^/Mn^2+^	Ratiometric fluorescence	Not specified	Not specified	Dual-emission probe using antenna effect	82
S,N-co-doped Nb_2_C MQDs	Cu^2+^	Fluorescence sensing	Not specified	2 µM	Also applied in cell imaging	83

In addition, the ion selectivity observed in different MQD-based sensing systems originates from several structural and chemical factors associated with the quantum dots. The specificity toward particular metal ions is largely governed by the composition of the MQDs, the type of heteroatom dopants, and the surface functional groups introduced during synthesis. Heteroatom doping can tailor the electronic structure and create defect-rich active sites that preferentially interact with certain metal ions through charge transfer or coordination processes. At the same time, surface functional groups such as amino, hydroxyl, sulfur-containing, or carboxyl moieties provide coordination environments with different binding affinities for specific ions. The synthetic methodology also plays an important role because it controls particle size, defect density, dopant distribution, and surface termination of MQDs. Consequently, variations in synthesis conditions and surface chemistry lead to distinct host–guest interactions with target ions, enabling MQD sensors to achieve selective detection of different metal ions across various sensing platforms.

### 4.6. Selectivity engineering in multi-ion environments: competitive binding, masking strategies, and signal discrimination

While MQD-based sensors have demonstrated excellent sensitivity toward individual transition-metal ions, maintaining reliable selectivity in complex multi-ion environments remains a critical challenge. Environmental, food, and biological samples typically contain multiple metal ions with comparable coordination behavior, including Fe^3+^, Cu^2+^, Zn^2+^, and Mn^2+^. Under such conditions, selective recognition cannot rely solely on the intrinsic affinity between a single ion and the MQD surface but instead emerges from the overall sensing strategy and chemical environment.

One widely adopted approach involves masking strategies, where specific reagents selectively complex interfering ions and reduce their effective participation in the sensing process. By suppressing competing interactions, masking agents reshape the ionic environment encountered by MQDs and improve discrimination toward the target analyte.^78–80^ This strategy reflects the practical reality that many transition metals exhibit overlapping coordination chemistry, making perfectly exclusive binding difficult to achieve at the material level.

Another emerging route involves differential signal modulation. In these systems, different metal ions produce distinct optical responses, such as fluorescence quenching, enhancement, wavelength shifts, or the generation of secondary emission bands. Rather than relying on a single binary response, MQD probes can therefore generate response patterns that allow discrimination between ions based on their optical fingerprints.

Although simultaneous multi-ion detection using MQD platforms is still relatively limited, these strategies provide practical routes for operating MQD sensors in complex ionic matrices. Approaches such as masking, ratiometric normalization, sequential sensing protocols, and controlled reagent addition can collectively improve analytical selectivity. Consequently, selectivity engineering is increasingly viewed not only as a property of the MQD material itself but also as a function of the overall sensing workflow and measurement design.

### 4.7. Toward deployable MQD-based sensing platforms: portability, visual readouts, and application-oriented integration

A growing direction in chemical sensing research is the transition from laboratory-optimized probes toward sensing platforms that can operate in practical environments. For heteroatom-doped MQD-based sensors, this transition involves extending performance evaluation beyond conventional analytical metrics such as detection limits and linear ranges. Increasingly, factors including portability, operational simplicity, visual interpretability, and stability under variable conditions are becoming essential for practical deployment.

Visual and semi-quantitative detection strategies represent an important step toward field-applicable sensing. In several MQD-based systems, fluorescence responses can be translated into colorimetric or visually discernible outputs, enabling rapid screening without sophisticated instrumentation.^79–82^ Test-strip formats, paper-based analytical devices, and simple fluorescence readouts have been explored as preliminary approaches for on-site detection, demonstrating the potential of MQDs for portable sensing platforms.

At the same time, achieving reliable performance under real environmental conditions requires robustness against fluctuations in pH, ionic strength, temperature, and sample composition. Such robustness is often achieved through system-level design, including protective matrices, internal referencing strategies, and controlled sensing environments. These developments highlight that practical sensing performance depends not only on the MQD material itself but also on the overall integration of the sensing platform.

Although fully deployable MQD-based sensing devices are still limited, current studies illustrate early steps toward portable and application-oriented systems. Continued integration of MQDs with simple analytical formats, portable detection tools, and application-specific workflows is expected to facilitate their transition from laboratory demonstrations to practical sensing technologies for environmental monitoring, food safety assessment, and bioanalytical screening.

### 4.8. Critical assessment and remaining challenges in MQD-based metal ion sensing

Despite the rapid progress in heteroatom-doped MQD platforms for transition-metal ion detection, several fundamental challenges remain that limit their broader analytical deployment. One persistent issue concerns the reproducibility of MQD synthesis. Variations in precursor chemistry, etching conditions, and post-synthetic functionalization can lead to significant batch-to-batch differences in particle size, defect density, and surface termination. These structural variations directly influence fluorescence behavior and ion-binding affinity, potentially affecting analytical reliability.

Another challenge involves selectivity in complex matrices. While many MQD sensors demonstrate excellent selectivity under controlled laboratory conditions, real environmental and biological samples often contain multiple competing ions, organic molecules, and fluctuating physicochemical parameters. Under such circumstances, nonspecific interactions or matrix-induced fluorescence perturbations may reduce analytical accuracy.^48–50^ Consequently, strategies such as ratiometric detection, masking agents, and internal referencing are increasingly employed to mitigate interference effects.

Long-term stability also represents an important consideration for practical applications. MQDs may undergo gradual surface oxidation, ligand detachment, or photobleaching during prolonged storage or repeated measurements. These processes can alter fluorescence intensity and sensing response over time, highlighting the need for improved surface passivation and protective matrices.

Finally, although MQD-based sensors have achieved impressive detection limits in laboratory studies, translation into scalable and field-deployable devices remains limited. Integration with portable sensing formats, standardized synthesis protocols, and robust validation in real-world samples will be essential for advancing MQD technologies toward routine environmental and bioanalytical monitoring. Addressing these challenges will help bridge the gap between promising laboratory demonstrations and reliable practical sensing systems.

### 4.9. Comparison of MQD-based sensors with other fluorescent nanosensors

Fluorescent nanosensors based on carbon dots, graphene quantum dots, semiconductor quantum dots, and metal–organic frameworks have been extensively explored for metal ion detection due to their tunable optical properties and high sensitivity. Carbon and graphene quantum dots typically exhibit good water solubility, low toxicity, and stable photoluminescence, which makes them attractive for environmental and biological sensing. Semiconductor quantum dots provide strong fluorescence emission and narrow emission bands, enabling highly sensitive detection; however, concerns regarding toxicity and long-term environmental impact have limited their broader analytical applications.^47,53^ Metal–organic framework-derived fluorescent systems also offer high surface area and adjustable coordination environments, which can enhance analyte interaction and signal transduction.

Within this broader landscape, MXene-derived quantum dots present a distinct class of fluorescent nanomaterials characterized by abundant surface terminations, strong electron transfer capability, and versatile chemical tunability. These features facilitate efficient interactions with metal ions through surface functional groups and defect sites, often leading to pronounced fluorescence quenching or enhancement responses. Compared with many conventional nanosensors, MQDs can provide rapid signal transduction and flexible surface modification through heteroatom incorporation, enabling selective recognition of specific metal ions.

Despite these advantages, MQD-based sensing systems are still developing compared with more established fluorescent nanomaterials. Several studies have reported excellent sensitivity and promising selectivity toward targeted ions, yet systematic comparisons across different nanomaterial platforms remain relatively limited.^49–81^ Therefore, placing MQD-based sensors within the broader family of fluorescent nanosensors helps clarify their emerging role, highlighting both their unique advantages and the areas where further optimization and comparative evaluation are still required.

## 5. Critical perspectives and future directions: scalability, selectivity limits, and real-world deployment of MXene QD-based sensors

MQDs have emerged as a versatile platform for transition-metal ion sensing due to their tunable surface chemistry, heteroatom-doping potential, and unique photophysical properties. Despite significant advancements in laboratory-scale demonstrations, several critical challenges remain for translating MQD-based sensors into practical environmental, biomedical, and industrial applications. Addressing these challenges requires a multifaceted perspective encompassing synthesis scalability, fundamental limits of selectivity, and integration into real-world deployment strategies.^48,78^

In addition to these broader perspectives, several fundamental bottlenecks continue to limit the practical implementation of MQD-based ion sensing systems. One key challenge is batch-to-batch reproducibility during MQD synthesis, where slight variations in precursor composition, etching conditions, or heteroatom incorporation can significantly alter particle size, defect density, and surface functional groups. These structural variations often translate into inconsistent fluorescence responses and ion-binding behavior, complicating quantitative sensing. Another concern is long-term stability. MQDs may undergo gradual oxidation, surface ligand rearrangement, or photobleaching during storage and repeated measurements, potentially leading to signal drift over time. Furthermore, although many MQD probes demonstrate excellent selectivity under controlled laboratory conditions, their performance can deteriorate in complex matrices containing competing metal ions, organic ligands, and fluctuating physicochemical parameters. These limitations highlight the importance of standardized synthesis protocols, systematic stability evaluation, and robust selectivity engineering to ensure reliable analytical performance in practical sensing environments.

A major constraint in current MQD research is the scalability of synthesis. Most high-performance sensors rely on hydrothermal, ultrasonic, or chemical exfoliation protocols optimized for milligram-scale production. While these approaches produce monodisperse, heteroatom-functionalized MQDs with high quantum yields, their translation to gram- or kilogram-scale quantities remains underexplored. Batch-to-batch variability, precursor costs, and reaction reproducibility are significant hurdles. For instance, nitrogen-doped Ti_3_C_2_ MQDs achieve ultralow detection limits for Fe^3+^ through surface redox interactions, yet the reproducibility of amino-functionalization in large batches remains uncertain. Emerging strategies, such as continuous-flow hydrothermal reactors or automated ultrasound-assisted synthesis, offer potential routes for scale-up while maintaining particle uniformity and surface chemistry fidelity. However, systematic studies quantifying the trade-offs between throughput, quantum yield, and doping efficiency are still scarce.^64,80^ Addressing these synthesis bottlenecks is essential for enabling industrial-scale production and widespread deployment of MQD-based sensors.

Equally critical is the fundamental selectivity limit of MQD systems. While heteroatom doping—such as N-, S-, or co-doping—provides tunable coordination sites for target ions, real-world samples present highly complex ionic matrices. Competitive binding, ion pairing, and nonspecific interactions can significantly alter fluorescence responses, particularly for chemically inert ions like Zn^2+^ or Mn^2+^. Traditional strategies often rely on masking agents, ratiometric normalization, or sequential detection protocols to enhance selectivity. Yet, these approaches have inherent limitations: masking agents may themselves interfere under multi-component conditions, and ratiometric readouts may not fully compensate for subtle matrix effects. Furthermore, the chemical design of MQD surfaces often faces trade-offs between selectivity and quantum yield, where extensive functionalization can quench emission or destabilize colloidal suspensions.^71,82^ A critical future direction involves integrating computational design and machine-learning-assisted screening to predict optimal heteroatom arrangements and binding motifs. This would allow rational tuning of surface sites for maximal selectivity against specific ion panels, rather than relying solely on empirical optimization.

In addition to machine-learning-assisted materials screening, density functional theory (DFT) calculations can provide critical atomic-level insights into the sensing behavior of heteroatom-doped MQDs. Computational studies can elucidate how dopant identity, concentration, and spatial distribution influence electronic band structures, charge-density redistribution, adsorption energies, and metal-ion binding affinity. For example, DFT-derived density of states (DOS) analysis may reveal how N- or S-doping introduces localized electronic states near the Fermi level, thereby facilitating charge transfer interactions with transition-metal ions such as Fe^3+^ or Cu^2+^. Similarly, adsorption-energy calculations can help identify preferential ion-binding sites and predict selectivity trends under competitive conditions. Integrating DFT analysis with experimental fluorescence and electrochemical observations would significantly strengthen mechanistic interpretation and support the rational design of MQD sensing architectures with optimized selectivity, stability, and electronic performance.

Beyond the current strategies, several material-level approaches could further enhance the sensing performance of MQD-based platforms. A particularly promising direction involves precise engineering of heteroatom distribution and defect structures within the MQD lattice. Instead of relying on random doping, future research could focus on controlled co-doping or the creation of single-atom active centers that generate highly selective coordination environments for specific transition-metal ions. Such atomic-scale design can significantly strengthen charge-transfer interactions between MQDs and target ions, leading to amplified fluorescence modulation and improved analytical sensitivity. Additionally, systematic control of MQD size distribution, edge termination, and surface passivation may enhance quantum yield and photostability, both of which are critical parameters for achieving ultralow detection limits. Integrating computational modeling and machine-learning-guided material design could further accelerate the discovery of optimized MQD structures with tailored electronic properties and selective binding motifs.

At the sensing architecture level, future research should also prioritize the development of advanced signal-transduction strategies that overcome the limitations of conventional single-intensity fluorescence quenching. Ratiometric fluorescence systems, fluorescence lifetime-based sensing, and FRET-mediated energy transfer platforms can significantly improve analytical accuracy by minimizing background interference and environmental fluctuations. Moreover, constructing hybrid nanostructures—such as MQD–metal nanoparticle, MQD–polymer, or MQD–MOF composites—may enhance sensitivity through synergistic electronic and plasmonic interactions. These innovations could enable multiplexed ion detection and real-time monitoring in complex matrices. In this context, integrating MQD sensing elements with portable analytical devices and IoT-enabled platforms may facilitate automated data acquisition and continuous environmental monitoring, transforming MQD-based probes from laboratory demonstrations into intelligent sensing systems capable of large-scale, real-world deployment.

Operational robustness and matrix adaptability constitute another essential axis for real-world deployment. Laboratory-based demonstrations often use buffered aqueous solutions or minimal ionic backgrounds, which do not reflect the variability encountered in drinking water, industrial effluents, or biological fluids. MQD performance under varying pH, ionic strength, temperature, and organic content is highly sensitive, potentially leading to false positives or signal drift. Recent trends highlight dual-emission ratiometric probes, sequential “off–on–off” signal modulation, and test-strip formats as promising strategies for maintaining reliable readouts across variable conditions.^56,78^ However, systematic evaluation under standardized protocols is limited. Future research should establish performance benchmarks that incorporate environmental variability, mimicking real matrices to assess robustness and reproducibility comprehensively.

Future research on MQD-based ion sensing should move beyond general performance improvement and focus on several concrete development directions. One promising approach is the controlled engineering of MQD surface terminations and heteroatom doping to regulate charge transfer interactions with specific metal ions, enabling more predictable sensing responses. Another important direction involves designing MQD composites with polymer matrices, porous substrates, or microfluidic platforms to improve probe stability and facilitate integration into portable sensing devices. Additionally, systematic studies that correlate MQD structure, surface chemistry, and fluorescence behavior are needed to clarify sensing mechanisms and guide rational sensor design. The development of standardized synthesis protocols may also improve reproducibility and allow more reliable comparison between studies. Finally, expanding investigations toward complex environmental and biological matrices will be essential for validating the practical applicability of MQD-based fluorescent probes under realistic analytical conditions.

A critical frontier lies in deployable sensor architectures that bridge laboratory performance with field applicability. Portable platforms, including lateral-flow devices, microfluidic chips, and smartphone-assisted fluorescence readers, are gaining attention. Visual readouts derived from colorimetric conversion of MQD fluorescence or ratiometric dual-emission systems offer low-cost, user-friendly options suitable for decentralized monitoring. Integrating MQDs with solid supports, polymer matrices, or paper-based substrates can improve colloidal stability and facilitate practical handling. Nevertheless, maintaining sensitivity and selectivity during immobilization remains challenging, particularly when heterogeneous matrices alter diffusion kinetics or binding equilibria.^80,83^ Advances in surface engineering, encapsulation chemistry, and device integration are therefore critical to achieve scalable, reliable, and user-oriented detection platforms.

Biocompatibility and multifunctionality represent an additional dimension, especially for applications intersecting biomedical imaging and ion detection. Certain Nb_2_C- and Ti_3_C_2_-based MQDs demonstrate dual utility in live-cell imaging and metal ion sensing, highlighting their potential for simultaneous diagnostic and monitoring applications. However, the long-term cytotoxicity, metabolic clearance, and biodistribution of heteroatom-doped MQDs remain largely unexplored. Future studies must systematically quantify biocompatibility while optimizing emission efficiency, surface passivation, and functional stability. The convergence of sensing and imaging applications could redefine the scope of MQD technologies, enabling simultaneous environmental and biological monitoring in a single platform.

From a broader perspective, data-driven integration and multiplexing will likely dictate the next generation of MQD-based sensors. The ability to discriminate between multiple ions within complex matrices using multidimensional fluorescence readouts, combined with real-time analytics, offers a path toward smart sensing networks. Pattern recognition, fluorescence lifetime mapping, and FRET-based ratiometric encoding could enhance selectivity and provide simultaneous multi-ion detection.^77,81^ Furthermore, combining MQD sensing platforms with IoT-enabled devices could allow automated, continuous monitoring, providing actionable environmental or biomedical insights.

While heteroatom-doped MQDs demonstrate exceptional promise for selective transition-metal ion detection, translating laboratory performance into practical, scalable, and reliable technologies requires addressing several critical challenges: scalable synthesis, fundamental selectivity limits, environmental robustness, device integration, and multifunctional applicability. Advances in automated synthesis, rational surface design, ratiometric and sequential signaling, portable device engineering, and smart analytics collectively define the future trajectory of MQD-based sensors.^79,82^ By systematically integrating these strategies, the field can progress from high-performance proof-of-concept studies to next-generation analytical tools capable of real-world environmental monitoring, biomedical diagnostics, and industrial quality control, positioning MXene QDs as a cornerstone of emerging quantum-dot-based sensing technologies.

## 6. Conclusion

Heteroatom-doped MQDs have demonstrated remarkable versatility and performance in selective transition-metal ion sensing, spanning Fe^3+^, Cu^2+^, Zn^2+^, Mn^2+^, and beyond. Through strategic surface engineering, including nitrogen, sulfur, or co-doping, MQDs can convert metal–ligand interactions into highly sensitive and tunable fluorescence responses. Redox-active ions, such as Fe^3+^ and Cu^2+^, benefit from quenching or ratiometric signal amplification, while chemically inert ions, such as Zn^2+^ and Mn^2+^, can be monitored *via* fluorescence enhancement or dual-emission ratiometric mechanisms. These diverse transduction modes expand the analytical scope of MQDs, enabling performance in complex matrices, environmental water, food samples, and even cellular imaging contexts.

Critical challenges remain in scalability, matrix adaptability, and real-world deployment. Laboratory-optimized MQDs must overcome synthesis reproducibility limitations, maintain selectivity under competitive binding conditions, and integrate into portable, user-friendly devices for field applications. Future research should emphasize rational heteroatom placement, multidimensional signal interpretation, and system-level engineering, including test-strip, microfluidic, and IoT-integrated platforms. Overall, MQDs represent a next-generation sensing platform that unites sensitivity, selectivity, and multifunctionality. By addressing current limitations and leveraging material diversity, MQD-based sensors are poised to transition from proof-of-concept studies to robust, deployable analytical tools, with broad implications for environmental monitoring, water quality assessment, and biomedical diagnostics. Their tunable optical properties, modular design, and adaptability firmly establish heteroatom-doped MQDs as a leading technology in advanced ion sensing research.

## Conflicts of interest

The authors declare that they have no known competing financial interests or personal relationships that could have appeared to influence the work reported in this paper.

## Data Availability

This article is a review and does not include any new experimental data. All data discussed and analyzed are derived from previously published studies, which are appropriately cited in the manuscript.
